# Using Digital Health to Enhance Risk Communication During Climate Events

**DOI:** 10.1055/s-0046-1816617

**Published:** 2026-11-03

**Authors:** Hania Rahimi-Ardabili, Kalissa Brooke-Cowden, Enrico Coiera

**Affiliations:** 1 Centre for Health Informatics, Australian Institute of Health Innovation, Macquarie University, 2109, NSWAustralia

**Keywords:** health communication, digital health, consumer health informatics, precision public health

## Abstract

**Objectives** : Risk communication is a critical component of health disaster management. Adverse health impacts are reduced by promoting the adoption of health-protective behaviours by health consumers. However, the success of risk communication relies on its ability to influence human behaviour and is shaped by perceptions, message relevance, and social and cultural influences. This perspective offers insights into how digital tools could support consumers during natural disasters by facilitating effective risk communication and addressing behavioural barriers.

**Methods** : This work examines factors influencing risk communication through the lens of human behaviour by consumers and introduces standard practices in risk communication. Digital solutions to climate-related risk communication are then proposed, drawing on existing evidence, real-world examples and authors' expertise to provide contextual understanding and practical viewpoints.

**Results** : Digital tools, by gathering and synthesising data, can assist in generating and disseminating tailored actionable messages to communities and debunking misinformation, particularly to vulnerable populations. Additionally, technologies that enable visualisation, simulation, sensing, and social connection can enhance risk understanding and create opportunities for the adoption of protective behaviour among consumers.

**Conclusions** : Leveraging digital technology using participatory approaches that consider consumer needs and involve consumers in developing these technologies, is likely to substantially enhance health care efficacy and success.

## 1. Introduction

Health risk communication to the general population is a crucial component of disaster management [ 1 2 ]. Health risk communication can involve timely information exchange about health risks arising from environmental or human factors to different at-risk audiences [ 2 3 ]. Effective risk communication can help avoid adverse health effects during health crises such as infectious disease outbreaks. The recent COVID-19 pandemic demonstrated the crucial role of risk communication in promoting health protective behaviours ( *e.g.* , social distancing) and reducing viral spread [ 4 5 6 ].

In the context of climate change, effective risk communication can prepare those at risk to protect themselves from climate change-related events such as extreme heat [ 7 ] and floods [ 8 ]. Yet decision-making, particularly in the context of emergencies, is complicated, and not all risk communication may lead to behaviour change [ 2 3 ]. Further, spreading misinformation and conspiracy theories during disasters can significantly undermine public trust in communications from governmental and health agencies. This issue was evident in the recent hurricane Milton in Florida, USA. Frequently spread, false accusations that the Federal Emergency Management Agency prevented Florida evacuations and diverted funding to undocumented migrants, demoralised first responders and complicated response efforts [ 9 ]. This article explores the current state of risk communication, examining the factors and barriers influencing consumers' responses to risk communication. It then discusses potential digital solutions with examples to enhance the effectiveness of risk communication during climate events.

## 2. Methods

This article employed a narrative, theory-informed approach to examine challenges and solutions in health risk communication for climate-related events. The authors synthesised relevant peer-reviewed literature and guideline documents for risk communication from authoritative bodies ( *e.g.* , WHO) to explore behavioural drivers influencing risk communication effectiveness.

The Behaviour Change Wheel (BCW) framework was used as a conceptual lens to identify and classify behavioural barriers and facilitators using its three domains of capability, opportunity, and motivation. This framework guided the mapping of communication challenges and potential digital health interventions. This comprehensive theory is derived from a review of 19 theoretical models, including the key models that consider individual, social, and environmental factors.

Real-world examples, including digital innovations and case studies in climate and health emergencies, were selected to illustrate how technology could be applied to support or improve consumer responses to health risks. These examples were chosen based on their relevance to identified challenges, practical significance, and geographic or contextual diversity. The authors' expertise in health informatics, health behaviour, and climate change was drawn upon to identify relevant technologies and contextualise the discussion.

## 3. Results and Discussion

### 3.1 Current state and barriers in risk communication for climate events

The most commonly used method of risk communication is a generic warning broadcast across mass and social media [ 10 11 ]. Yet such communication can be inefficient and fail to reach target populations - leading to suboptimal behaviour changes and may be considered inconsistent or untrustworthy [ 8 12 13 ]. A failure to address the specific needs of a population can lead consumers to view such communications as redundant [ 14 ] and disregard health warnings or see them as generic and disconnected from their lived experiences, especially in vulnerable communities [ 12 13 ]. Resistance to change may be due to consumer struggles with multiple/interacting health, social, psychological and financial stressors [ 14 ].

Communication is most effective when tailored, actionable, and based on vulnerability [ 2 15 ]. Messages should be specific about how individuals can stay safe. Such risk communication can mitigate the adverse effects of climate-related health crises [ 3 13 ]; however, they are not sufficient on their own. Multiple factors drive human behaviour beyond awareness and knowledge. According to the BCW theory, human behaviour adoption is only possible when all three main drivers of capability, opportunity, and motivation are met at physical, psychological, and social levels [ 16 ]. Capability refers to an individual's psychological and physical ability to perform a behaviour, including their skills, memory, knowledge, and physical resources. Opportunity encompasses the external factors and resources, including social influences ( *e.g.* , misinformation and social roles) or environmental conditions. Motivation relates to internal drivers, including an individual's desires, beliefs, the perceived value of the behaviour and emotion. Motivation can stem from adaptive conscious thought processes or reflective unconscious mechanisms [ 16 17 ]. Figure 1 maps risk communication problems in climate-related events across three main behavioural drivers [ 16 ] alongside potential digital solutions, which will be discussed in the next section.

**Figure 1.**
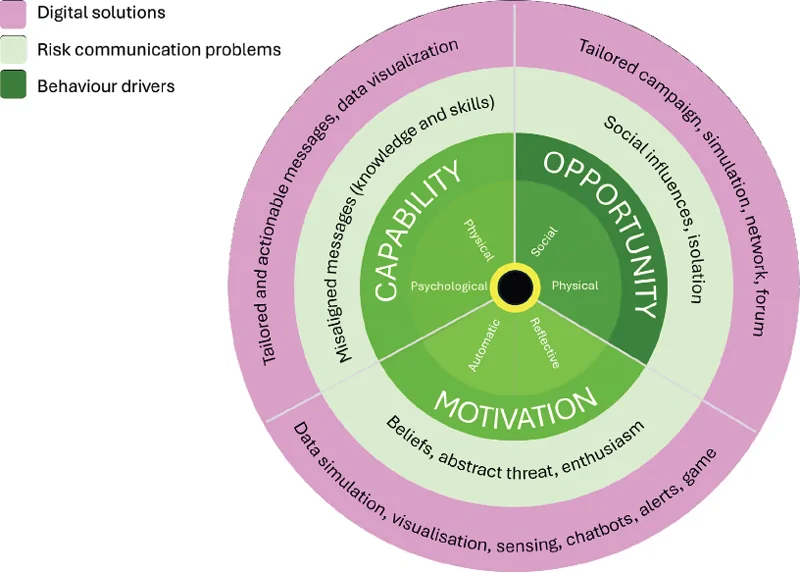
Risk communication problems and digital solutions categorised by three behaviour drivers adapted from the Behaviour Change Wheel [ 16 ].

For example, a social media comment analysis showed that, in aggregate, the Phoenix community in the USA exhibited an underwhelming behavioural response to heat warnings during high temperatures [ 18 ]. Normalisation of heat conditions as routine rather than a threat, limited warning effectiveness and curbed residents' behavioural response. In this scenario, identity and a sense of belonging to the community served as barriers to adopting protective behaviour (lack of social opportunities) [ 18 ]. Another example of human behavioural drivers could be the perceived likelihood of events or health risks [ 19 ]. A study showed that homeowners' likelihood to take protective behaviour against bushfires is influenced by their perceptions of risk severity, self-efficacy, and response efficacy [ 20 ]. Perceived likelihood of events can act as a barrier as climate threats may seem abstract, distant or difficult to imagine [ 21 ]. Behaviour adoption is more likely to fail when the threat is perceived as a potential future challenge instead of an imminent and credible threat [ 22 ].

One of the key barriers to health behaviour adoption is misinformation, which presents a critical challenge in risk communication, particularly during health disasters [ 23 ]. During disasters, the production of massive amounts of data from multiple sources of varying quality may have significant social and health-related impacts [24]. Digital communication platforms play a significant role in spreading accurate and inaccurate information, and digital illiteracy further fuels misinformation [25]. An umbrella review of 17 systematic reviews found that misinformation can distort public understanding, exacerbate fear, and hinder the adoption of effective responses [26]. Misinformation not only undermines trust in official communication channels but also disrupts resource allocation, delays care provision, and amplifies societal divides [26]. Therefore, strategies addressing misinformation should be a pivotal part of risk communication.

Various resources have highlighted the importance of a tailored approach and human behaviour drivers, specifically in the context of risk communication. WHO's risk communication guidelines emphasise the importance of trust-building, consumer engagement, and message design [ 2 ]. WHO's strategic communications framework also specifies six principles for effective risk communication: accessible, actionable, credible, trusted, relevant, and timely. It further reinforces the influence of consumer awareness, perceived risk, perceived response efficacy, confidence, and social/cultural norms [27].

### 3.2 How digital health can be part of the solution

Digital health technologies provide a powerful means to enhance risk communication in various ways ( Figure 1 ). Here, we offer various digital solutions that fulfill the six components of human behaviour drivers related to climate change that must be addressed for risk communication to succeed. Table 1 summarises examples of potential digital solutions for each problem type.

**Table 1.** Problems and example solutions for risk communication and promoting protective health behaviour during climate events (Behaviour drivers adapted from Michie et al. 2011 (16), with the remaining being the authors' conceptualisation)

Behaviour drivers	Problems/barriers	Digital solutions	Digital tasks
Capability -psychological	Misaligned messages with consumer needs (knowledge)	Customised, tailored, actionable messages	Communication, decision support system
Capability -physical	Reduced mobility	Digital platform for connection and building a climate-related social care network	Communication
Opportunity -physical	Lack of resources and access issues	Resource mapping apps	Decision support system
Opportunity -social	Social influences e.g., norms, misinformation, trust	Visual graphics using generative AI Community engagement by trusted figures through customised online forums Prompts on the accuracy of information in social media	Communication, decision support system
Motivation -reflective	Perceived risk and response efficacy Self-efficacy	Risk assessment via sensing, e.g., alerts on vital signs by wearables Virtual coaches, e.g., empathetic chatbots Games and simulations for practising in safe and realistic environments, with awards	Communication, sensing, decision support system
Motivation -automatic	Abstract threat	Data simulation and risk visualisation of consequences, e.g., virtual reality that evokes emotions	Decision support system

One approach that digital solutions can assist with in risk communication is by tailoring the message and providing timely and actionable steps for those who need it most. For example, automated phone warnings targeting at-risk populations in real-time may enhance risk awareness. Mehiriz *et al.* , in two randomised controlled trials, demonstrated that this approach promoted health-protective behaviours and/or reduced healthcare use during extreme heat and smog episodes [28,29].

The message design dashboard (MDD) is another digital decision support tool for framing and customising risk communication messages. Launched by the US Federal Emergency Management Agency, the MDD uses rule-based automation to support alert authorities. It provides templates based on input variables related to the specific hazard to draft effective and tailored warning messages informed by behaviour theories [30]. MDD is designed to draft messages that enhance consumer understanding, belief, and self-efficacy, ultimately enhancing consumer responses [31].

Beyond templates, digital technology, by aggregating data from various resources can help to develop customised messages based on consumers' medical conditions, housing, socio-economic status, location, and weather data similar to the individualised marketing approach, which is increasingly being used to tailor advertisements to improve marketing efficacy and engagement [32]. Such a system could refine its responses through feedback loops, integrating new data to improve message impact. Applying such principles in public health emergencies could support authorities in delivering more targeted and effective risk communication.

The role of digital health in risk communication extends beyond immediate responses to climate events. It also encompasses long-term approaches for promoting protective health behaviours. Advanced digital technologies are important in facilitating the three main human behaviour drivers [33] (see Figure 1 ).

One approach involves utilising immersive virtual reality, to support knowledge acquisition and belief development in an engaging and gamified environment, addressing the capability and motivation drivers of behaviour change. It can enable users to visualise health consequences or have a virtual “lived experience” of climate events, with the advantage of enhancing risk understanding [34]. For example, virtual reality risk communication technology which simulates flood scenarios to assist with flood risk understanding, showed that those who underwent the simulation were more likely to invest in flood preparedness than those who did not. It might also contribute to skill development and self-efficacy (capability driver) without imposing any risk, by practising in a safe and interactive environment while receiving feedback on user performance, improving confidence and optimism [35]. In a gamified risk communication study, users walked through a virtual environment maze with clear dangers and different types of warning signals. This allowed users to interact with the environment and test the consequences of their actions, helping them better understand risks and making lessons more memorable [33]. Gamification, by promoting a sense of achievement with badges and points, has the potential to motivate people to adopt health-protective behaviours, such as preparing for extreme weather events by maintaining an emergency preparedness kit [36].

Promotional campaigns addressing social influences, such as norms (social opportunities), could benefit from the wide reach and accessibility of digital channels and data mining features to deliver customised and tailored messages aligned with community group values. These approaches could create impactful interventions to influence communities' understanding and awareness of individual risks. Further, recent advancements in generative artificial intelligence (AI) could enable clear, emotionally resonant, and culturally relevant content—both written and visual—that align with community values and enhances tailored messaging [37]. This is especially valuable in emergencies, where AI can adopt an authoritative and humanistic tone [38], and create a sense of urgency and ensure additional accessibility through integration into chatbots and virtual assistants. Generative AI could foster engagement and enhance the trustworthiness of communication by combining well-crafted content [37] with design elements such as colour, imagery, logos, and style [39]. Further, AI generated imagery can improve conceptualisation of complex climate change narratives which may otherwise present as abstract issues [40].

Digital technologies can provide mechanisms to counter misinformation at the system level. One application could be real-time quality control over communication platforms, enabling automated monitoring and identification of false information. This could facilitate targeted resource-smart interventions and regulations. For instance, machine learning could track multiple cases of misinformation, including incorrect details, fabricated emergency warnings, false news or misleading visuals on X (formerly Twitter) during various disaster events, such as Hurricane Harvey [41], achieving a high accuracy rate in distinguishing between true, false, and neutral information. By flagging misinformation early, panic and confusion among health consumers could be reduced, and authorities may use this information to better understand and counter misinformation more effectively.

Digital technologies could further facilitate literacy, as a component of public education initiatives, through educational campaigns and social media platforms, equipping health consumers with the skills to assess online information critically and tackle misinformation. A systematic review of 26 digital interventions against COVID-19 misinformation identified various digital interventions, including accuracy nudges ( *e.g.* , prompts warning on the reliability of particular online information), prebunking (strategies helping consumers identify misinformation before exposure), debunking (correcting false claims), and gamification (by promoting critical thinking) [42]. These approaches, when combined with co-designed educational campaigns delivered through social media or apps, may foster more informed and resilient consumers [42]. Similar to risk communication strategies, framing digital literacy messages to audiences' specific values, concerns, and beliefs might increase communities' receptiveness to accurate information and reduce the impact of misinformation during critical events [43].

However, technologies are not without limitations. Notably, generative AI has been associated with environmental costs due to its high computational and intense energy use of generative AI; therefore, this highlights the need for more thoughtful and transparent integration of AI, with developers aiming for net-zero greenhouse gas emissions when designing projects, and authorities or organisations responsible for implementation considering strategic and greener options for deployment [44,45].

Despite the potential of digital technologies to enhance communication, their effectiveness can be limited by disparities in internet access and resource barriers. To address these challenges, it is essential, where possible, to complement digital channels with alternative non-digital communication methods [46] and to implement infrastructure transformation such as expanding broadband access (especially in underserved and rural areas) and policies addressing system barriers ( *e.g.* , insufficient digital devices) [47]. Further, digital platforms should be designed to consider accessibility and inclusion especially in less resource-intensive settings, ensure functionality in low-bandwidth settings and compatibility with assistive technologies such as screen readers and voice recognition software, to support equitable access for all consumer groups [46].

### 3.3 What's next in designing successful digital interventions

#### 3.3.1 Understanding consumer needs

While digital tools have the potential to support health risk communication during climate-related events by addressing behavioural barriers, the success of such interventions centres on understanding consumer needs. Despite the health consequences of climate change being evident, their full scale and impact, particularly among those with multiple vulnerabilities [48] and for certain under-represented climate-sensitive diseases, are yet to be fully examined [48,49]. The interconnected effects of climate change on environmental, social, and economic systems make health needs complex and context-dependent [50]. Consumer perceptions and responses to climate change are also poorly studied and pose challenges to success [48,49].

Better data integration and disease-sensitive surveillance could facilitate exploring the unmet needs of consumers and monitoring changes by capturing real-time data on consumer experiences and vulnerabilities [51]. For instance, mining social media data can assist with disease monitoring or understanding consumer perception and responses to certain climate events. A study by Vicari *et al.* serves asvan example of risk perception exploration and changes in patterns within tweets about the 2016 Seine River flood (Paris) in a broader disaster risk context [52]. This exploration of consumers' perceptions can be applied to the health discipline to identify vulnerabilities. The information gathered from consumer responses during climate events provides valuable insights for refining future risk communication and disaster management strategies [51]. Analysing this data helps identify what worked well and what needs improvement, informing ongoing efforts to enhance preparedness and response. This iterative process of learning and adaptation is essential for building more effective digital interventions and improving overall disaster management.

#### 3.3.2 Designing participatory interventions

A participatory approach at every intervention stage, from design to implementation, is the best practice for addressing consumer-specific needs [53,54]. This approach amplifies consumer engagement, enhances compliance, and fosters a sense of ownership and agency [55]. Various dimensions need to be considered. One example could be considering preferred communication channels [ 15 ]. Different communities have varying needs and concerns regarding how information is delivered. Digital literacy and their ability to identify misinformation, fraud concerns, and generational preferences all play a role in shaping these preferences [56]. Therefore, it is essential to customise communication channels to match the needs of consumers, particularly among those who are vulnerable, ensuring that messages are accessible and engaging.

## 4. Conclusion

Digital health technologies offer hope in the face of climate change, providing innovative solutions to enhance risk communication and promote protective health behaviours during climate crises, particularly by supporting and targeting vulnerable communities. Box 1 summarises future actionable steps to strengthen health consumer resilience in response to climate crises using digital technology. Digital tools combined with consumer involvement could enable the delivery of personalised, relevant information and debunk misinformation to foster resilient communities for climate-related health crises. As we navigate the complexities of a changing climate, digital health stands as a powerful ally in our quest to protect and empower communities.

**Box 1.** Future actions to build resilient health consumers by leveraging technology for climate crises.

Future Actions
• Identify the consumers' unmet needs and their climate responses through real-time disease monitoring and data integration from various sources. • Develop tailored digital risk communication interventions, especially targeting vulnerable consumers. • Utilise digitally empowered consumer preparedness approaches informed by human behaviour drivers of capability, opportunity and motivation. • Leverage digital technology to target misinformation by detecting timely false narratives, debunking them, and promoting digital literacy. • Consider a participatory approach at every stage of interventions, from design to implementation, to ensure consumer voice, values and needs.
